# “I just wasn’t expecting it”: lived experiences of early menstruators and the impact of parental support in competitive dance

**DOI:** 10.3389/fspor.2025.1688876

**Published:** 2025-11-24

**Authors:** Alysha Ambrosio, Margo E. K. Adam

**Affiliations:** 1 Faculty of Rehabilitation Medicine, University of Alberta, Edmonton, AB, Canada; 2 Faculty of Kinesiology, Sport, and Recreation, University of Alberta, Edmonton, AB, Canada

**Keywords:** early menarche, competitive dance, adolescent development, menstrual experiences, parental support, period management, youth athletes

## Abstract

**Introduction:**

Menarche is a significant milestone in adolescence, yet the implications of early menarche [menarche before 12.72 years (Canadian mean)], remain insufficiently understood. Early menarche is generally linked to increased mental health risks, which may be intensified by the appearance-based pressures of competitive dance. Existing literature emphasizes menstrual disorders and delayed menarche, leaving early menarche in dance largely underexplored

**Methods:**

This study investigated how young women in competitive dance perceive and experience early menarche, and how parental support influences dancers' ability to express needs and navigate this transition. Five dancers (Mage = 14.62) who experienced their first menstruation at 11.58–12.54 years (M = 12.24), participated in one-on-one semi-structured interviews, and five parents joined a focus group. Guided by interpretive description, data were analyzed using reflective thematic and functional analysis.

**Results:**

Three themes were generated to reflect athletes' experiences: Alone in the Spotlight: What It Means to Menstruate Early, Behind the Curtain: Presence and Absence of Support, and Dancing Through Discomfort: Symptoms, Struggles, and Strength. Two shared dancer-parent themes were generated: Step by Step: Mastering the Art of Hiding Periods and Choreographing Comfort: Medication for Period Symptoms. Three additional themes were generated to reflect parent experiences: The Prep Step: Early Education and Preparation Before Onset, Backstage Support: Helping Young Dancers Manage Their Period, and Dad's Got the Moves: Stepping Up for Her Cycle.

**Discussion:**

Findings from this research highlight the unique, multi-faceted challenges of early menarche in competitive dance and underscore the need for tailored education, support, and resources for dancers and their families.

## Introduction

Menarche, a young woman's first menstruation, marks the onset of reproductive capacity and serves as a key milestone in female pubertal development (1). While early menarche is widely acknowledged [Canadian mean age of 12.72 years (2)], its broader psychosocial and developmental implications remain critically underexplored. Harris et al. (3) describes a 0.73-year decrease in the age of menarche across birth cohorts, signaling a long-term trend toward earlier onset among Canadian girls. Similarly, Wang et al. (4) reported a decline in the mean age at menarche in the United States, from 12.5 years among the 1950–1969 birth cohorts to 11.9 years among the 2000–2005 birth cohorts. The timing of menarche is shaped by multiple influences and social and psychological stressors have been shown to accelerate pubertal development through physiological pathways (5). Early menarche has been associated with a range of adverse outcomes, including depression, anxiety, excessive psychosomatic symptoms, eating disorders, metabolic syndrome, obesity, substance use, truancy, and poor academic performance (2, 6). Walvoord (7) suggests that the early onset of these biological and social transitions increases the likelihood of maladaptive coping mechanisms, while Allison and Hyde (5) emphasize the role of hormonal shifts in impacting arousal, stress sensitivity, and emotional regulation.

In sport, young women frequently normalize menstrual discomfort, dysfunction, and irregularities as a natural part of being an athlete (8). Menstruation is often framed as a nuisance, with physical symptoms interfering with training and impairing performance (8, 9). Psychological effects, such as decreased motivation, low energy levels, anxiety, and irritability, further impact athlete well-being and performance (8, 10). These difficulties, coupled with the challenges of early menarche, can be detrimental to an individual's experience and continued participation in sport. This is particularly concerning given the well-established benefits of sport participation, including enhanced self-esteem, improved social skills, reduced symptoms of depression, and increased confidence and competence (11). Addressing the challenges menstruation poses, particularly for early menstruators, is thus essential for fostering more inclusive and supportive athletic environments.

Dance is often categorized as an aesthetic sport, characterized by harmony of movement, artistic expression, and athleticism (12). According to Dwarika and Haraldsen (13), the dance environment is “unrelenting” marked by constant pressure to perform and conform to narrowly defined physical ideals. Appearance plays a pivotal role in performance evaluation, placing dancers at heightened risk for mental health concerns, including anxiety, burnout, and disordered eating (12, 13). These pressures may be especially acute for early menstruators, whose pubertal transitions unfold in public, body-conscious environments that value uniformity, control, and poise (14). Although awareness of menstrual health in dance has grown, existing literature focuses predominantly on menstrual dysfunction or delayed menarche (15–17). Yet, within competitive dance—where tight-fitting costumes, rigorous performance expectations, and an implicit standard of bodily uniformity prevail—early menstruators may be particularly vulnerable to feelings of exposure, shame, and physical discomfort (14, 18). Euphemistic language, secrecy, and the normalization of discomfort reinforce a culture of silence that marginalizes menstruating bodies, even in spaces that claim to promote self-expression and empowerment (8, 19, 20).

Parental support during menarche plays a vital role in shaping young women's experiences, yet it remains inconsistently addressed in the literature. Research highlights that communication around menstruation often lacks depth, clarity, and emotional support, especially when menarche occurs earlier than expected (21, 22). The absence of informed and empathetic parental involvement may limit a young dancer's capacity to voice needs, manage discomfort, or seek guidance, thereby heightening feelings of isolation and anxiety (21, 22).

This study addresses these gaps by exploring the lived experiences of early menarche among young women in competitive dance, and the role of parental support in shaping their ability to navigate this transition. The purpose of this research is to explore and describe the nuanced ways early menarche is experienced and managed within the unique sociocultural context of competitive dance. Our research is seeking to present a more holistic understanding of the challenges faced by early menstruators, and to provide recommendations for more responsive practices in dance education, healthcare, and parenting.

## Materials & methods

### Design

This study employed interpretive description methodology to examine early menarche experiences in competitive dance, aiming to identify common themes while accounting for individual variation in participants' perspectives (23, 24). Prior to data collection, the researchers engaged in reflexive practice to clarify their positionality and potential influence on the study. The design was informed by both theoretical and practical knowledge and refined through ongoing reflection on assumptions (23). Researcher–participant interactions were acknowledged as shaping the perspectives of both parties, and reflections were shared with participants to foster rapport and create a safe environment for discussion. Guided by interpretive description, the methodology enabled the identification of themes and patterns in subjective perceptions to generate insights that both deepen understanding and have practical application (24).

### Recruitment and participants

Following ethics approval, participants were recruited using purposeful, convenience, and snowball sampling strategies through established connections in the Canadian dance community. Recruitment efforts included posters, advertisements, and in-person visits to an urban dance studio where research assistants had established relationships. This familiarity fostered trust and supported the creation of a safe environment, which was essential for participants to engage with the study's inherently vulnerable subject matter. Interested individuals and/or their parent(s)/guardian(s) were invited to contact the research team and complete an eligibility screening survey via a secure Qualtrics survey. Only individuals who met the inclusion criteria were invited to participate. Our goal was to recruit four to five athletes along with at least one parent or guardian per athlete in order to generate rich, interconnected data. The study sought to achieve transferability, allowing findings to be applied to comparable contexts such as other aesthetic sports (e.g., gymnastics and figure skating). Sample sufficiency was evaluated during the interview process through attention to data saturation, with recruitment continuing until no new insights emerged.

Five young women 12–16 years of age (*M* = 14.62) who began menstruating before the Canadian mean age (*M* = 12.24 years), participated in this study (see Table 1). All participants had engaged in competitive dance during the onset of menarche and were currently dancing. Competitive dance was defined as participation in at least one competition within the past twelve months, along with a minimum of five hours of weekly training in competitive, technical dance styles: ballet, contemporary, jazz, hip-hop, lyrical, modern, tap, or acrobatics. Parents and/or guardians of these dancers were also recruited to participate in the study.

**Table 1:** Dancer ages at interview by menarche by years of competitive dance experience.

Dancer	Age during interview (years)	Age of menarche (years)	Competitive dance experience (years)
Emma	14.75	12.17	5.00
Kylie	13.58	12.51	6.00
Grace	13.67	12.54	6.00
Laura	14.16	12.42	6.00
Diana	16.92	11.58	9.00
	*14* *.* *62*	*12* *.* *24*	*6* *.* *40*

Note: Italicized entries reflect sample means.

To protect confidentiality, the research team assigned pseudonyms to all participants.

### Data generation

The study employed semi-structured individual interviews with young dancers and a semi-structured focus group with the parents and/or guardians to explore lived experiences related to early menstruation within the context of competitive dance. Each dancer participated in one in-person, semi-structured interview conducted at their dance studio to enhance participant comfort and encourage open dialogue. Interview questions were designed to explore participants' experiences of early menarche, their menstrual knowledge and challenges, sources of support, and the social dynamics about menstruation in dance environments. Parents and/or guardians of the participating dancers were invited to take part in one sixty-minute semi-structured focus group. The focus group explored their understanding of menstruation, the forms of support they provided during their child's developmental transition, and strategies used to address menstrual health within the competitive dance context. All interviews and focus groups were audio recorded with informed consent, and field notes were taken by research assistants to capture contextual details, non-verbal cues, and observations that might enrich the subsequent analysis (interview and focus group guides are available upon request).

### Data analysis

Data were analyzed using two complementary qualitative approaches: reflective thematic analysis (25) and functional analysis (26, 27). Audio recordings from interviews and focus groups were first manually transcribed verbatim and reviewed multiple times to ensure accuracy and develop familiarity with the data. Key ideas, narratives, and notable quotes were highlighted across transcripts. These excerpts were then organized into logical groupings aligned with recurring patterns, from which central themes were identified. The initial themes included: early menstruation, symptoms and symptom management, period management and fears in dance, support systems, period knowledge, perceptions of menstruation, and body image. Within each theme, related quotes were grouped into subthemes to enhance clarity and conciseness, and subthemes were summarized to support a deeper understanding of participants' experiences. Once subthemes were refined, the overall thematic structure was reviewed, and central ideas were distilled to ensure clarity and coherence. Functional analysis further contributed to understanding how participants used language, structure, and emphasis to convey meaning and emotion. Eight themes were developed to reflect the patterned meanings across the dataset in relation to the research questions: three themes represented the experiences of dancers, three captured perspectives from parents, and two illustrated overlapping insights. These themes function as descriptive categories and as analytic tools that illuminate how participants made sense of their experiences within competitive dance. Three athlete-centric themes, two parent-centric themes, and two crossover themes were generated and are described and discussed.

## Results & discussion

### Alone in the spotlight: what It means to menstruate early

Many young women have limited, vague, or negative knowledge before menarche, leading to confusion and fear when menstruation begins (28). Early menarche before emotional or educational readiness can cause distress and isolation. For instance, *Kylie* initially mistook her first period for “a stomach ache.” Similarly, *Emma* recalled thinking, “Oh my gosh, did I pee myself? … I put on a liner… I didn't know what else to do because my mom wasn't there.” *Laura* also expressed uncertainty, turning to her mother with the question, “What's going on? Like, what do I do?” These narratives reflect not just limited biological knowledge but a deeper absence of embodied understanding. Consistent with these findings, Rubinsky et al. (22) reported that some individuals viewed menarche as a sign of severe illness or imminent death, underscoring the urgent need for comprehensive, supportive, and anticipatory menstrual education. Some participants, like *Grace*, demonstrated a basic biological understanding: “It's like when a girl becomes a woman, so you can get pregnant in the future… one of the eggs comes out.” In contrast, others—*Laura*, *Kylie*, and *Diana*—expressed uncertainty, offering hesitant or incomplete explanations. *Emma* remarked, “I don't really know how to explain it … it's kind of just like when you bleed once a month … you're not pregnant.” Collectively, these perspectives underscore the fragmentary and inconsistent nature of early menstrual knowledge. Menstrual knowledge itself is multidimensional, encompassing biological understanding, menstrual dysfunction, and practical management skills (29). Participants often demonstrated one without the other, and when compounded by stigma and limited dialogue, these gaps may hinder the development of comprehensive, actionable knowledge. Such foundational understandings of menarche shape how young women interpret and manage menstruation, influencing not only coping strategies but also broader self-perceptions. This connection between menstrual knowledge and body perception is particularly significant in dance. Danis et al. (30) found that although most dancers achieved a desired body mass index, many remained dissatisfied with their body image—a factor that may elevate the risk of mental health concerns (12, 13). In this context, early and unexpected menstrual experiences can leave lasting impressions, as menstruation is closely tied to identity, maturity, and self-concept (31).

When asked whether they felt prepared for menarche, participant's responses varied considerably. *Diana* was frank, “No … I had no idea when I would get it.” In contrast, *Kylie* felt “pretty ready”. *Emma*, though unprepared, remained unfazed: “It didn't make me uncomfortable. I was just like, okay, like, this is just a thing that's happening to me now.” *Grace* offered a mixed response, “Yes and no…I was a little surprised … I didn't really want to get it,” while *Laura* simply said, “Yeah,” but added, “I was annoyed.” These varied responses illustrate the unpredictability of early menarche and the broad spectrum of emotional and informational readiness among young women. In line with these findings, Marván and Molina-Abolnik (32) reported that only 39% of participants felt prepared for menstruation. Additionally, experiencing menstruation at an earlier age can be a significant stressor itself, heightening the risk of adverse psychological outcomes—especially when individuals face menarche without adequate support or education (5, 22, 33). *Diana* exemplified this, recalling, “I was really stressed.. I didn't really know how to deal with it.” Hormonal changes associated with early menarche may further intensify sensitivity to stressors, compounding vulnerability to psychological distress (5). These risks are intensified in dance, where athletes face a unique combination of training-specific demands, performance expectations, and psychosocial pressures that are often disproportionate to opportunities for recovery. Such cumulative stressors can undermine physical health, psychological well-being, and performance readiness (34). Collectively, these intersecting pressures place dancers at heightened risk for psychological distress and related adverse outcomes.

Early menstrual education is often inconsistent and stigmatized (21, 22). In the present study, participants described varied experiences with menstrual education that, notably, excluded explicit reference to dance. This absence suggests that the specific challenges of managing menstruation in dance contexts were minimized or disregarded, leaving dancers without guidance tailored to their embodied realities. *Kylie*, *Diana*, and *Grace* learned primarily through school-based programs, while *Emma*, whose school lacked sexual education due to COVID-19, learned from her mother and personal experience. *Laura* credited maternal openness at age eight or nine. These accounts reflect broader findings that many young women receive fragmented, emotionally unsupported information—often through impersonal formats like videos or classroom instruction—leading to confusion, discomfort, and internalized stigma (21, 22). For example, *Diana* recalled, “We watched a video, and then [the teacher] had a question box,” while *Kylie* noted, “I've probably, like, heard of [periods], but I didn't exactly know what they were when I was younger … Grade four, sex ed we learned about periods.*”* Similarly, *Grace* reflected, “I kind of knew before, but I really found out in a sex ed class.” Brown et al. (29) further highlight this issue, revealing that young women aged 10–15 in England and Wales lacked awareness relating to numerous aspects of the menstrual cycle and identified a substantial deficit in school-based menstrual education, particularly in relation to strategies for managing periods while maintaining physical activity. Such gaps not only leave young women underprepared for practical challenges but also reinforce a culture in which menstruation is hidden, stigmatized, and inadequately addressed in environments critical for physical and psychosocial development. These shortcomings in formal education are mirrored in participants' experiences. In contrast to school-based learning, *Emma* described a more gradual and relational learning process through her mother: “I didn't know it existed until my mom first talked to me about it. So I kind of just learned, like, what it was and then I gradually just, like, learned more stuff about it as I got older.” This aligns with Costos et al. (35), who found that while 50% of participants identified their mothers as their first source of information, the majority reported negative messaging (64%) and limited openness, with 68% unaware of their mothers' own menstrual experiences. Such findings suggest that although mothers often serve as primary educators, their guidance is frequently shaped by silence and stigma, constraining opportunities for open dialogue and embodied learning. In Rubinsky et al. 's study (22), participants who experienced early or unexpected menarche, received reactive, directive, and narrowly focused support, leaving both young women and their caregivers unprepared. In response to these limitations, Bellas et al. (36) emphasized the need for menstrual education that is introduced earlier, developmentally appropriate, and inclusive of all genders. Such education should be clear, supportive, and aligned with young people's cognitive and emotional development (36).

Participants in this study similarly underscored the need for straightforward, age-appropriate education. *Grace* explained it helps “you know what's happening with your body and you don't get scared,” while *Emma* added, “So that you don't freak out and be like, am I dying?” *Diana*, *Laura*, and *Kylie* echoed this need, emphasizing that such information helps young women “know how to deal with it,” “what to do,” and “what's, like, actually happening.” Despite early discomfort, most participants eventually came to see menstruation as natural. *Kylie* said, “It's just… normal. I'm still me.” *Grace* reflected, “I kind of knew it was normal… everybody gets their period.” *Emma* added, “It's a little inconvenient… but it's just natural.” This gradual normalization reflects findings by Burrows and Johnson (37), who observed that young women often reframe their understanding of menstruation over time, particularly when supported by open communication. For early menstruators, this process is especially important, as they frequently undergo physical changes before their peers and may feel socially “out of sync”, especially within the dance environment, which places a strong emphasis on uniformity (14, 38).

This study shows that early menarche is often marked by fear and confusion, intensified by stigma, vague language, and detached instruction. Many participants were unprepared—both practically and emotionally—for this transition. The findings highlight the need for early, inclusive menstrual education that normalizes varied timelines, addresses emotional and physical experiences, and offers clear, supportive context-specific guidance. With proper support, early menarche can be met with confidence instead of fear.

### Behind the curtain: the presence and absence of support

“I just wasn't expecting it … I was kind of in shock. I kind of panicked and didn't ask anybody … I was in my ballet stuff … I didn't really know how to deal with it. So I just kind of put some shorts on and went home.”—*Diana*

*Diana's* reaction to early menarche—shock and silence—reflects the unpreparedness many young menstruators face, where asking for help feels out of reach. She later admitted she “probably could have asked” someone but did not know how. Emotional responses to menarche vary widely and are shaped by the quality of communication: affirming, shame-free conversations empower young women, while silence or judgment deepen confusion and reinforce shame (21, 22). *Grace* emphasized the need for normalization: “Just like, more… how it wasn't embarrassing. Because, like, everybody gets their period.” This reflects Rubinsky et al.'s (22) finding that young people want menstruation presented as a common, manageable experience that still acknowledges its emotional and physical realities. Furthermore, effective menstrual education must encompass the diverse experiences of dancers, including early menarche, delayed menarche, and menstrual dysfunction, to ensure that young women are both prepared and empowered. These insights underscore the need for open, supportive conversations that affirm both the normalcy and complexity of menstruation.

In the absence of broader social support, maternal involvement becomes essential. Most young menstruators turn to their mothers for information and reassurance—echoing Marván and Molina-Abolnik's (32) finding that 94% discuss menstruation with their mothers before menarche. *Kylie's* memory captures this bond: “When I got home, I was, like … oh, I gotta get my mom,” she recalled, adding, “My mom prepared me for that stuff… She was like, oh, okay, this is what you do.” This highlights the mother's role as a trusted source of both practical guidance and emotional comfort during early menarche. These dynamics reflect broader gendered expectations in dance, where mothers are disproportionately involved in their children's activities and often positioned as endlessly available facilitators, while fathers typically occupy more limited or passive roles (39). This unequal distribution of responsibility reinforces traditional caregiving norms and places a heightened burden on mothers.

This exclusion persists in dance and in menstruation management despite evidence that many fathers are both knowledgeable and willing to engage (31, 40). Participants rarely confided in their fathers about menstruation. When asked, *Laura* replied bluntly, “No.” *Kylie* said she would only speak to her father “if it's just me and [my dad],” and *Emma* noted, “My dad kind of just, like, knows that it's not really his thing.” These responses suggest paternal support is typically seen as a last resort, not a primary source of comfort or information. *Diana's* earlier story at the studio, when her mother was away, further illustrates this gendered divide:

“I called [my mom], and I didn't tell my dad. He got really mad at me. He was like, why didn't you tell me? I was really embarrassed. But then it was fine. Then my mom called him and told him what was wrong. I was kind of ignoring him. That was it.”

This highlights how excluding fathers from menstrual conversations limits opportunities to reduce stigma and build shame resilience—roles increased paternal involvement could help fulfill (41, 56). However, most men have limited and often inaccurate menstrual knowledge, shaped largely by media, outdated education, or female partners (40, 42). These sources often reinforce secrecy rather than empathy, and when combined with fathers' reduced involvement in dance activities, menstruators become less likely to view male figures as accessible sources of support (39, 41, 56).

Nonetheless, some participants reported support from both parents, suggesting that when fathers engage with sensitivity, their involvement can foster a more open and affirming environment. At the same time, early menstruators often navigate the tension between seeking support and asserting independence. *Emma* voiced concerns that too much parental support “would be a little overbearing,” while *Laura* struggled to articulate what support she needed— highlighting how support needs are individual and evolving. Expanding menstrual conversations to include all caregivers, regardless of gender, can help dismantle stigma and foster more informed, supportive experiences, while still respecting young women's autonomy as they learn to manage these changes on their own terms.

Even with care and support at home, being the first among peers to experience menarche can heighten feelings of disorientation and isolation, especially without relatable social support (38). *Diana* recalled, “Most of my friends hadn't gotten their periods yet, so I couldn't really ask anyone,” and *Emma* said, “I had no friend who was like, oh, I know what you're feeling,” highlighting the emotional solitude early menarche can bring. However, as they matured, peer support became a vital source of affirmation. *Emma* explained, “I know that the people around me have got my back,” and *Laura* noted, “[My friends] are going through many of the same experiences… so if I need help, they can help me.” These accounts underscore the importance of shared understanding in normalizing menstruation and encouraging physical activity, as sport participation has been shown to enhance social skills, self-esteem, and confidence—factors critical to sustaining long-term engagement (11, 43). At the same time, Walker et al. (44) identified both personal and environmental factors contributing to dance drop-out, including training demands, high expectations, loss of passion, and challenges in forming social connections. Peer support can serve as a protective factor in this context; however, depending on the social environment, it may also generate anxiety (37).

Overall, intentional support from parents and peers can boost confidence, ease anxiety, and encourage openness. *Grace* and *Kylie* said it made them feel “more prepared” and that “it makes it a little bit easier.” *Laura* found support helped her talk openly about menstruation, while *Emma* said it created “a safe space to ask questions.” These reflections show that even when early menstruators do not clearly express their needs, having trusted people and accessible resources provides essential emotional safety. Sometimes, even passive support—like a family member noticing mood changes—can help normalize menstruation and reduce isolation. In sum, the quality, accessibility, and emotional impact of support are crucial. Open, informed, and nonjudgmental guidance empowers young women to face menstruation confidently. Yet silence, stigma, and gender norms often leave early menstruators to cope alone highlighting the need for inclusive, practical support systems involving families, peers, and studios to reshape the menstrual experience.

### Dancing through discomfort: symptoms, struggles, and strength

Early menstruators often face unfamiliar and disorienting physical and emotional symptoms that complicate their adjustment to puberty (45). Vichnin et al. (46) reported that 86% of adolescents experience menstrual pain, with 39% facing moderate to severe disruptions in daily activities. Participants in this study described a range of symptoms—including cramps, back pain, nausea, fatigue, and imbalance—that varied monthly; cramps were universal. For dancers, even mild discomfort can disrupt coordination and balance, crucial for a discipline demanding precision and aesthetic control (12, 47, 48). *Diana* and *Kylie* described feeling “off their leg” or “off balance,” while *Emma* recounted severe symptoms: “My cramps were like 9 out of 10… I ended up throwing up… I actually missed dance for it.” This highlights how menstrual symptoms can hinder participation, interrupt training, and impair performance (8). *Diana* also linked her cycle to recurring hip tightness, showing how menstruation can compound existing physical challenges. The unpredictability of symptoms adds complexity; *Grace* described her cramps as “not too bad,” while *Emma* said, “Some months I'm fine… sometimes the cramps are too much that I can't move.” This variability makes preparation and self-management especially difficult in high-performance settings.

Menstruation also impacts psychological well-being, causing low energy, irritability, and decreased motivation that can undermine confidence and performance (8, 10). *Diana* said, “I feel kind of gross… I'm not working my hardest,” while *Laura* described feeling “less energy… more drowsy, like, groggy.” These experiences mirror findings by Burrows and Johnson (37), who noted many young women use illness-related language—pain, fatigue, mood swings—to describe menstruation. *Emma* shared, “If I'm not feeling it that day, I'll usually just not come to dance,” showing how menstruation, even when normalized, can reduce motivation and comfort. Yet sport participation brings significant physical, psychological, and social benefits (11). This contrast reveals the tension between menstruation as a natural process and its experience as a source of vulnerability. Without proper support, this perception risks reinforcing stigma by portraying menstruating bodies as weak rather than resilient and capable. Furthermore, hormonal fluctuations across the menstrual cycle can strongly affect emotions, behaviour, and social interactions—boosting confidence during ovulation but causing fatigue, anxiety, and depressive symptoms in the luteal phase—shaped by biological, psychological, and environmental factors (49). Participants reported varied emotional shifts: *Emma* said, “I tend to cry more… I was just trying not to,” despite her instructor's focus on dance technique. Others felt “grumpy,” “more frustrated,” or “annoyed easily,” often without understanding why. For early menstruators, these changes can be particularly confusing and stigmatizing, as many peers have yet to experience menstruation and its challenges.

In sports, young women often normalize menstrual discomfort, dysfunction, and irregularities as part of being an athlete, which can obscure the need for appropriate support (8). The pressure to perform despite pain or fatigue is especially evident in competitive settings. *Laura* explained, “In a competition, I just, like, go for it. … adrenaline kind of, like, keeps me, like, going.” *Diana* noted, “On stage, If I'm kind of focused, I don't really notice it.” Yet this determination often hides unspoken discomfort. *Kylie* summed it up: “I kind of just deal with it,” and *Emma* added, “I'll just suck it up and go for it.” This normalization reflects a cultural narrative where menstruation is endured silently—managed but not discussed—leaving many without the language, tools, or peer support to advocate for themselves, especially in athletics where menstruation is minimized or treated as private. Keil et al. (10) found athletes face limited institutional support, inadequate education, and persistent stigma, reinforcing menstruation as a solitary burden rather than a shared concern.

Described as “unrelenting,” dance imposes intense pressure to perform and conform to narrow aesthetic ideals, with bloating, discomfort, and early physical changes exacerbating body image concerns due to the central role of appearance in self-perception and evaluation (12, 13, 50). *Laura* shared, “I feel less confident … Because I get, like, bloated on my period, so I just, like, feel, like, ugh.” *Emma* reflected, “Going through puberty… that has impacted my image more than my period,” pointing to the layered challenges of adolescence where menstruation intersects with broader self-image shifts. Early menarche can exacerbate these challenges by causing visible changes before peers, increasing self-consciousness (5). Schooler et al. (50) also linked negative attitudes toward menstruation to greater body image concerns and discomfort. Encouragingly, Silva et al. (51) found that healthy movement, such as moderate to vigorous physical activity, may reduce depressive symptoms associated with early menarche, suggesting that continued physical activities like competitive dance can be protective with proper support.

Overall, early menstruators face a dual burden: managing complex, fluctuating symptoms while navigating environments that often overlook their needs. Meaningful emotional and practical support can transform this experience from isolating to empowering. Recognizing menstrual health as central to youth well-being does not hinder excellence in dance, it sustains it.

### Step by step: mastering the art of hiding periods in dance

In competitive dance, standout performances rely on audience appeal, technical skill, musicality, and physical appearance (18). At the study's studio, intermediate and senior dancers must complete at least 6.25 h of ballet weekly to qualify as “competitive,” with participants typically averaging 7–7.5 h. Ballet's social culture emphasizes bodily uniformity, favoring specific shapes, sizes, and movement qualities while discouraging physical differences (14). Within this environment, early menstruation introduces physical and emotional challenges that heighten feelings of difference.

Tight-fitting attire like high-cut leotards and ballet tights, combined with fear of leaking and pressure to maintain an ideal aesthetic, intensifies self-consciousness (8, 10). Rubinsky et al. (22) found many participants recalled early menstruation marked by actual or feared leakage, showing how deeply these anxieties embed during adolescence. *Grace* expressed, “I just obviously hope I don't bleed through my tights or something,” while *Emma* shared: “I've also seen, like, girls bleed through their tights and I'm like, that is not happening to me. No way.” Anxiety often peaks during performances, with *Grace* noting, “I might be more nervous on stage … anybody could see it and it's not just my friends.” Menstrual irregularities, common among dancers due to intense physical exertion (52), add further complexity. *Emma* shared, “I actually didn't get [my period again] for, like, 120 days. … [My doctor said] it's normal to have irregular periods if you exercise so much.” Similarly, *Grace* described hers as “not super consistent,” while *Kylie* noted, “sometimes I'll have it twice in a month, and some months I just won't have it at all.” This unpredictability can increase anxiety about leakage and preparedness.

Before learning to use tampons, dancers described discomfort using pads with tights. *Diana* recalled, “Wearing shorts over tights before you learn how to put a tampon in, cuz wearing a pad with tights is really uncomfortable.” *Emma* agreed: “It's not enough to wear, like, a light tampon … I don't want to wear, like, a pad with my, like, leotard and stuff.” For many, menstruating early in a dance environment is a deeply personal challenge played out in a highly public space, where any mishap feels amplified and exposing. Women often take physical and mental measures to hide menstruation—such as concealing products and avoiding disclosure to peers or instructors—which, though invisible to others, create unnecessary stress and anxiety that can hinder participation, strain relationships, and diminish daily experiences (19, 20). Dancers' strategies for managing and concealing menstruation reflect a constant negotiation between bodily comfort, social norms of discretion, and the physical demands of their craft. *Kylie* explained, “If I have pants for a costume, I'll usually just wear period underwear. But if I have a leotard, I'll probably use a tampon … tampons, I just use so that you can't really see them at all, and they won't peek out of leotards or costumes.” *Emma* described combining products: “I might wear a liner in my bodysuit or wear period underwear with a tampon so I don't have to worry about fiddling with the liner.” *Laura* noted that period underwear worked well “for dance, for, like, tights and stuff.” Malefyt and McCabe (42) found that women often adjust the absorbency and fit to prioritize comfort, but dancers must balance comfort with invisibility, selecting products that effectively conceal menstruation under performance attire. Outside performances, clothing choices also shift: *Diana* preferred joggers and hoodies during her period, while *Kylie* “usually I'll wear pants instead of shorts.” These adjustments highlight how menstruation shapes body awareness and self-presentation, echoing Burrows and Johnson's (37) findings that young women alter clothing to conceal menstrual signs.

While menarche is a natural aspect of female development, it is often surrounded by scrutiny, secrecy, and shame (20). Parents in this study described the emotional labor involved in supporting their daughters through menstruation within environments that demand discretion and bodily control. *Parent K*^1^ described her daughter's anxiety: “She didn't want to wear underwear in her … bodysuit and couldn't figure out how to like deal with it.” To help, she purchased thong-style period underwear: “She was very concerned … about people knowing … she thought everyone would care.” Other parents noted that even wearing shorts, a minor change, could signal menstruation and cause embarrassment. *Parent E* explained, “It wasn't common to wear the shorts in ballet,” and *Parent D* affirmed, “People knew when you were wearing the shorts what was going on.” These accounts reveal how young dancers and their parents grapple with an unspoken but deeply felt social code around menstruation.

In the midst of these challenges, leak-proof underwear emerged as a “dancer's savior,” “magical,” and even “teen saviors” in the eyes of several parents. *Parent K* called it “so much nicer. … It's just better and easier.” *Parent E* described it as “a saving grace,” enabling her daughter to dance without worry. Yet, finding the right products involved trial and error. *Parent K* recalled, “The ones I bought were not working,” while *Parent E* noted the challenge of finding the right size for her petite daughter. These experiences highlighted a broader gap in guidance and communication around menstruation in the dance world. As *Parent E* reflected,

“We're used to getting our list of, like, here's all the supplies you need for dance. … it would be so awesome if it was more open about here are some options for this type of stuff that you may or may not. You know, like, for types of underwear.”

These narratives show how dancers and parents quietly manage menstruation in a highly disciplined, visible, and body-conscious environment. Dancers feel pressure to stay composed and discreet, while parents provide practical support that protects both privacy and autonomy. Together, they co-construct strategies to maintain performance and confidence. Despite reassurances that “no one cares,” dancers often bear an internal burden shaped by dance's intense scrutiny, where any loss of control feels exposing. This unspoken coordination emphasizes the urgent need for open, inclusive, and practical conversations about menstruation in dance communities. Providing supportive tools and clear information is essential to reduce stigma and build confidence for both dancers and parents.

### Choreographing comfort: medication for period symptoms

When managing menstrual symptoms, dancers commonly rely on over-the-counter (OTC) medications, especially ibuprofen-based options like Advil. *Laura* simply said, “Um, Advil,” while *Grace* mentioned, “just, like, take Midol or Advil or something,” advice passed down from her mother. *Kylie* takes a proactive approach: “I mostly just take all the medicine before I go to dance.” *Emma* schedules her medication carefully, saying, “Taking Advil throughout the day … I love my Advil … for nausea I really like taking ginger pills.” *Diana* uses prescription medication when symptoms are severe. These accounts show both the physical challenges dancers face and their growing autonomy in managing symptoms. However, this reliance on self-medication raises concerns—Kelly et al. (53) found that fewer than one-third of adolescents knew ibuprofen was Advil's active ingredient, and many lacked awareness of side effects, dosages, and brand equivalents, underscoring the risks of unsupervised medication use in high-performance environments.

Parents, similarly, turn to medications as a primary tool to help their daughters manage mesntrual symptoms. When asked about the type of support they provide, whether emotional, educational, physical, social, or psychological, *Parent D* responded plainly, “Drugs.” *Parent K* elaborated, “She doesn't want to take [medication], and I'm like, trust me, you need to learn to take the drugs.” Medication was framed not only as pain relief but also as a life skill key to maintaining independence at school or dance. *Parent E* noted, “There's nothing [the school] can do if they don't bring their own [medication]” *Parent D* described her proactive planning: “I can put in the calendar that she should start taking it … The PMS symptoms are a lot less when we plan for them … Diana's missed dance because of PMS.” Malefyt and McCabe (42) similarly found that both mothers and daughters commonly use OTC or prescription medications to manage menstrual discomfort, underscoring symptom control as a shared, practical priority. Proper dosage often requires medical guidance; *Parent E* recalled consulting a doctor about adult Advil dosages, noting, “This 12 year old is not as big as … the average size of a 12 year old. So I remember being thankful that I asked.” In contrast, *Parent L* noted, “Laura has never asked for or taken any medication,” highlighting the varied impact menstruation has on dancers. For many, though, medication is essential to maintain physical, social, and emotional functioning.

Both dancers and parents view medication as a practical and often necessary tool. Dancers approach symptom management with growing confidence, while parents emphasize prevention and preparedness. Together, they build a shared care framework grounded in planning, education, and trust. This contrasts with findings from Rubinsky et al. (22), where many adolescents lacked validation or information when managing menstrual pain. Here, involved and communicative caregivers enhance dancers' experiences, underscoring the value of empathetic guidance. However, medication is not the only option. Management should be tailored to symptom severity, individual preferences, and medical history (54). For mild symptoms, education and reassurance may suffice, while nonsteroidal anti-inflammatory drugs like ibuprofen are recommended for others (54). Non-medication strategies—diet changes, physical activity, cognitive behavioural therapy, vitamin supplements, herbal remedies, and acupuncture—can also support well-being (54). These alternatives, alongside medication when needed, help dancers maintain participation and nurture overall health. Ultimately, equipping young dancers with knowledge, choices, and autonomy fosters not only symptom relief but also lasting confidence, resilience, and body literacy.

### The prep step: early education and preparation before onset

Many parents navigated their daughters' early menstruation with a blend of surprise, openness, and practical readiness. *Parent E* recalled the COVID-19 pause in school sex education saying, “Her teacher emailed parents saying, ‘We're not going to talk about sex ed this year so here's all the stuff about puberty you teach your kid, right?’ … it opened the door for conversation, which was really awesome.” Others emphasized the value of early, casual talks. *Parent K* shared:

“Kylie was curious … we just kind of talked about generally… when it first happens, it's kind of a nothing, but it's like, brown in your underwear, but it's not poop. And if you ever see that, come get me.”

*Parent G* contrasted this openness with her own upbringing:

“When I was younger, it wasn't a thing that you talked about… I even told her my experience, and I told her about even my friend's experiences. So it was something very normal and it wasn't something that we had to be quiet about.”

This intentional openness signals a generational shift—while older generations often viewed menarche as confusing or taboo (21), many modern parents aim to normalize menstruation and break longstanding silences (42).

Practical preparedness was another common theme. *Parent L* explained, “We made her like a little kit for her school bag … she started having cramps and stuff like that… we just had little kits everywhere.” *Parent K* took a similar approach: “I had all the packs of stuff—in school backpack, dance backpack … her little friend had it, and she was nine.” *Parent E* added that early periods were becoming more common in her daughter's peer group: “We also had this stuff in the backpack because a lot of her friends… were kind of going through that when she was in grade 6.” Despite this, early onset often came as a surprise. *Parent E* recalled: “It was summer right before grade 7. And I was surprised too because it was earlier than I had expected.” *Parent D* was also caught off guard:

“I was so shocked how early it came… I was between grades seven and eight. That's when my first period came. Diana was January of grade five. So I was like, oh my god, I'm not ready for this. It's not time… I don't think we really prepared her.”

These reflections reveal that although early menarche can be surprising, it often leads to greater openness, support, and practical preparedness. Many parents reframe menstruation education—providing the transparency, care, and guidance they themselves may have lacked. Early periods become an opportunity to build trust, reduce stigma, and nurture resilience through compassionate, informed parenting.

### Backstage support: helping young performers manage their period

Parents often described early, irregular menstruation as a major challenge in dance, where timing and control are crucial. “That first year or so it's not regular at all,” said *Parent E*. *Parent K* added, “No. You never know,” making pre-planning nearly impossible. Dancers, with maternal guidance, begin early cycle tracking to anticipate their periods and their needs, turning forecasting into invisible labour amid dance demands. These irregularities often feel disruptive and uncontrollable. To manage, families adopt proactive strategies—*Parent D* explained, “The PMS symptoms are a lot less when we plan for them.” Others, like *Parent G*, focus on open communication: “If she's ever sore or if she's in pain, [she will] let me know.” These approaches reflect a shift from traditional caretaking toward strategic partnership in parenting.

Parents learn to recognize behavioral and physical cues before young women can articulate them, with mothers attuned to a broader range of symptoms and fathers more often noticing mood swings and visible signs like heavy bleeding (55). *Parent L* noticed shifts in mood: “Her mood changes, but… she realizes, okay, maybe I'm being a bit of an asshole.” *Parent G* shared, “With Grace… I have no idea. She doesn't get cramps or anything.” *Parent E* highlighted exhaustion as an early indicator: “She'll say, ‘I'm super exhausted, and I just do not feel up to dancing tonight … You might say something else and all of a sudden she's in tears.” These subtle cues help parents support their daughters as they learn to understand and manage their changing bodies.

Tampon use emerged as a key, but often stressful, milestone. Malefyt and McCabe (42) found that mothers typically introduce pads first, as internal products tend to provoke anxiety. *Parent D* acknowledged the pressure to adapt quickly: “She figured it out really fast… being athletes… they have to manage it faster.” Before learning, *Parent K's* daughter was reluctant to attend dance: “She didn't want to come because she couldn't figure it out.” Stressing preparation, *Parent K* said, “The last thing you want is to get to competition and have that be your first time … You're nervous already.” She provided her daughter with both tools and autonomy: “I gave her the instruction book… She closed the door, and she figured it out.” For others, like *Parent E*, it was a gradual, shared experience: “We're just in the aisle… these are my go-to brands… now let's see what they have for teens … It wasn't for a while until she felt comfortable using a tampon.” For young dancers, learning tampon use is more than hygiene—it's a step toward bodily autonomy, confidence, and fitting into performance culture. Parents must navigate their own anxieties while supporting their daughters' growing independence.

### Dad's got the moves: stepping up for her cycle

Many parents reflected on the unique challenges fathers face when their daughters begin menstruating, especially in cases of early onset or maternal absence. For Laura's family, both parents serve in the Canadian Armed Forces, and her mother was deployed during interviews. *Parent L*, Laura's father, spoke candidly about navigating unfamiliar territory:

“My biggest fear was [her Mom] would be gone when this would have happened … Not a fear in like, how do I handle this? But I was like adamant … if you're gone away, this is gonna be a pad, this is not gonna be a tampon … I am not, as a dad, comfortable with putting a tampon in my 10-year-old.”

Despite his initial anxiety, he described feeling proud:

“I was actually excited to be home for that, I —strange enough— didn't want to miss it. We were like, oh, that's so amazing. We didn't make it like a … bad thing at all. … But it's definitely different for a dad like you have to be like, okay this is a totally different scenario.”

Other parents echoed this theme. *Parent D* shared how her husband stepped up during Diana's first period while she was away: “It made them have to talk about it. … the dad couldn't just sort of like … hide and never address it.” *Parent G* emphasized how openness eased tension: “She's very open about that stuff … with [me] and even with [her dad].” Still, for some, paternal discomfort remained a barrier. *Parent K* reflected on how discomfort can hinder communication:

“She'll phone me … she's very shy about talking, and I wish that [her dad] was a little less … ‘ew' … maybe she wouldn't be so, you know, dependent on me then.”

Others used humor to normalize shifting roles. *Parent E* laughed about her husband's growing awareness: “He will look at me like if she's having … “teenager-ness” and he's like oh is this like, you know.” These reflections highlight an evolving role for fathers. While initial discomfort is common, many are learning to meet their daughters' needs with support, curiosity, and growing confidence. This shift aligns with Girling et al. (55), who found that only 19% of fathers reported discomfort, while nearly half of daughters felt unsure about approaching them. Whether managing logistics, addressing emotional discomfort, or simply showing up with care, these fathers are learning to normalize—and in some cases, celebrate—this pivotal transition. In doing so, they build trust, deepen connection, and foster resilience during a critical stage of adolescent development.

## Limitations

This study has two limitations that should be considered when interpreting its findings. That all participants were recruited from a single Canadian competitive dance studio. This localized approach may reflect specific cultural, socioeconomic, or regional factors. Second, recruitment through existing networks may also have introduced sampling bias, as participants may have shared similar resources or support systems.

Despite these limitations, the study offers valuable insights into the experiences of early menstruators in high-performance dance and underscores the need for supportive, inclusive, and context-specific menstrual care.

## Future directions

Building on this research there are several avenues for further exploration and examination. Specifically, one area to explore is the the impact of early menstruation on sport drop out with intentional efforts being put toward tangible strategies and supports that can foster deep and accurate education for parents and coaches on how to manage this major life event. Another line of research to consider is the role that peers and coaches or instructors play in supporting young menstruators in dance.

## Conclusion

The experiences of early menstruators in competitive dance demonstrate a powerful intersection of embodiment, performance, and care. While early menarche can trigger confusion, fear, and stigma, it also presents opportunities for connection, education, and resilience—especially when met with empathy and intention. Menstrual care in high-performance settings extends beyond product use or symptom relief; it involves emotional labour, cultural expectations, and the unique pressures of the dance world. When families and communities respond with openness—offering tools, validation, and honest dialogue—menarche can become a moment of empowerment rather than vulnerability. Achieving this requires cultural reimagining of who participates in menstrual care. Involving fathers, coaches, and peers helping normalize menstruation in public, performance-based spaces like dance.

## Data Availability

Deidentified data will be shared by the authors upon request.

## Footnotes

1 To protect confidentiality, parent participants are referred to as "Parent X," with "X" corresponding to the first letter of their child's pseudonym.
